# Clinical Differences Between Urban and Rural Populations in Adolescents with Clinical High Risk of Psychosis

**DOI:** 10.1192/j.eurpsy.2025.2243

**Published:** 2025-08-26

**Authors:** P. Navalon Rodriguez, Y. Cañada, A. Jovani, A. Adriasola, L. R. Bofill, E. Pechuan, A. G. Blanco

**Affiliations:** 1 Psychiatry Department, La Fe Hospital; 2 Mental Health Research Group, La Fe Health Research Institute, Valencia; 3 Mental Health Institute, Hospital del Mar, Barcelona, Spain

## Abstract

**Introduction:**

Individuals at Clinical High Risk of Psychosis (CHR-P) show increased risk for developing psychotic disorders. The relationship between the risk of developing psychosis and urbanicity has been previously described; however, there are divergent results regarding the relationship between positive psychotic symptoms and urbanicity.

**Objectives:**

The present study aims to analyze the clinical and sociodemographic differences between an urban and a rural population of youth with CHR-P.

**Methods:**

The characteristics of the CHR-P program at La Fe Hospital (Valencia) are described and compared for the two populations in the study: a rural area comprising 10 towns with populations ranging from 200 to 29,000 inhabitants each, with an average of 12,600 inhabitants, and an urban area corresponding to the northern metropolitan area of Valencia. An analysis and comparison of the sociodemographic and clinical characteristics of the general population in both areas is also conducted.

**Results:**

Preliminary results are provided: The sample consists of 46 patients, 21 from the rural area and 25 from the urban area. The average follow-up for both groups was 8 months, with a transition rate to psychosis during this period of 19% (n=4) for the rural area group compared to 0% for the urban area group (p=0.04). Patients in the rural area group exhibited greater severity of positive psychotic symptoms with a higher positive PANSS score (14.19 ± 4.32) compared to the urban area group (11.12 ± 3.67), and this difference was significant (p=0.032). No statistically significant differences were found between the two groups for the rest of the variables.

**Conclusions:**

The preliminary results of our study show greater symptom severity in individuals from rural areas. Demographic factors, resource provision, or delays in care might be related to this finding.

**Disclosure of Interest:**

None Declared

